# Metamemory ratings predict long-term changes in reactivated episodic memories

**DOI:** 10.3389/fnbeh.2015.00020

**Published:** 2015-02-09

**Authors:** Amnon Yacoby, Yadin Dudai, Avi Mendelsohn

**Affiliations:** ^1^Department of Neurobiology, Weizmann Institute of ScienceRehovot, Israel; ^2^Sagol Department of Neurobiology, University of HaifaHaifa, Israel

**Keywords:** metamemory, reconsolidation, episodic memory, feeling of knowing, declarative memory

## Abstract

Reactivation of long-term memory can render the memory item temporarily labile, offering an opportunity to modify it via behavioral or pharmacological intervention. Declarative memory reactivation is accompanied by a metamemory ability to subjectively assess the knowledge available concerning the target item (Feeling of knowing, FOK). We set out to examine whether FOK can predict the extent of change of long-term episodic memories by post-retrieval manipulations. To this end, participants watched a short movie and were immediately thereafter tested on their memory for it. A day later, they were reminded of that movie, and either immediately or 1 day later, were presented with a second movie. The reminder phase consisted of memory cues to which participants were asked to judge their FOK regarding the original movie. The memory performance of participants to whom new information was presented immediately after reactivating the original episode corresponded to the degree of FOK ratings upon reactivation such that the lower their FOK, the less their memory declined. In contrast, no relation was found between FOK and memory strength for those who learned new information 1 day after the reminder phase. Our findings suggest that the subjective accessibility of reactivated memories may determine the extent to which new information might modify those memories.

## Introduction

Reactivation of long-term memories renders them transiently sensitive to potential long-term modifications via behavioral or pharmacological means in a process termed reconsolidation (Sara, 2008; Hardt et al., 2010; Dudai, 2012; Schwabe et al., 2014). This suggests a route to modify established memories, e.g., enhance desired recall (e.g., Coccoz et al., 2011; Forcato et al., 2011; Forcato et al., 2013), update information (e.g., Forcato et al., 2007; Hupbach et al., 2007), weaken memories (e.g., Schwabe and Wolf, 2009; Chan and LaPaglia, 2013), or block intrusive, traumatic recollections (e.g., Schiller et al., 2010; Agren et al., 2012; Oyarzun et al., 2012). In recent years, reconsolidation has been demonstrated in humans for both non-declarative (e.g., Walker et al., 2003; Censor et al., 2010; Kindt and Soeter, 2013) and declarative memory (e.g., Hupbach et al., 2009; Strange et al., 2010; Kroes et al., 2014). Evidence for alteration of declarative memories has been shown for verbal memoranda (Forcato et al., 2007, 2009b), lists of objects or pictures (Hupbach et al., 2007; Wichert et al., 2011), as well as more naturalistic memories such as movies (Chan and LaPaglia, 2013) and autobiographical events (Schwabe and Wolf, 2009; Kroes et al., 2014). Successfully destabilizing and rewriting the memory trace was reported to depend on a variety of conditions, among them the strength of the reactivated memory trace, the applied post-reminder manipulations, and the way reminders are presented (Hardt et al., 2010; Alberini, 2011; Schiller and Phelps, 2011).

A unique aspect of declarative memory retrieval is the person's ability to access in real time knowledge about the retrieval process (Tulving and Madigan, 1970). This faculty, referred to as “metamemory,” constitutes the capacity to reflect upon memories as they unfold and to evaluate their accuracy. A major metamemory capacity is “Feeling of Knowing” (FOK), referring to one's experience regarding the relevant information available for recollection, even if the retrieval attempt ultimately fails (Nelson and Narens, 1990; Koriat, 1993; Schwartz, 1994). FOK judgments are suggested to be formed based on the sense of familiarity of the retrieval cues and the information they target (Reder and Ritter, 1992). According to the *accessibility account* of FOK (Koriat, 1993, 1994), such judgments are formed on the basis of the vividness, specificity, and intensity of partial retrieved information, be it correct or incorrect, implying that FOK may signify the degree or strength of reactivated memories.

There is evidence to suggest that the intensity of reactivation influences the retrieved memory's vulnerability to subsequent manipulations (Alberini, 2011). Thus, highly reactivated memories brought about by prolonged exposure to reminders were shown to be more sensitive to post-reactivated administration of protein synthesis inhibitors (Suzuki et al., 2004), as well as memory traces that come to dominate behavior (Eisenberg et al., 2003). Recent studies in humans suggest that heightened degrees of memory reactivation, assessed by either subjective recollection (St. Jacques and Schacter, 2013) or by manipulating trace dominance of spatial memory (Bridge and Voss, 2014), corresponds to increased susceptibility to memory update. It is noteworthy that using memory tests as reactivation cues often enhance the probed memories (Carpenter et al., 2008; Karpicke and Roediger, 2008) and could render them resistant to post-test interference (Potts and Shanks, 2012). Conversely, reminders that consist of partial or contextual memory cues seem to contribute to reconsolidation of the original memories (Hupbach et al., 2007; Forcato et al., 2009a). Here we set out to test the hypothesis that the level of FOK assessment of episodic memory availability corresponds with the degree of memory change induced by post-retrieval manipulations.

Toward this end, we devised a protocol wherein participants watched a short movie episode, immediately followed by a memory test for items presented in the movie. One day later, immediately after being reminded through a partial retrieval cue of the movie episode and reporting their FOK for scenes depicted in the movie, they watched a second, unrelated movie (*Manipulation Group*). A separate group viewed the second movie without a preceding reminder and FOK assessment (*No reminder Group*). Still another group watched the second movie 1 day after the reminder stage, outside of the presumed reconsolidation window (*Delayed Manipulation Group*). On day 4, memory for the original movie was re-tested in all groups, and a differential memory performance score (“*memory strength*”) was calculated, representing changes in memory performance between the initial and final memory tests. We find that one's subjective assessment of memory availability during retrieval can predict the extent to which new information acquired immediately thereafter will modulate those memories. This could provide an immediate, accessible measure of the effectiveness of post-retrieval manipulations on the fate of reactivated memories.

## Methods and materials

### Participants

Seventy seven Hebrew speaking participants (26.4 ± 0.4 years old, 35 females) were recruited from a database of undergraduate and graduate students at the Weizmann Institute of Science and the Faculty of Agriculture of the Hebrew University, Rehovot, Israel. All participants asserted in a written consent form that they did not suffer from vision or hearing disabilities or learning and memory disorders. The experimental protocol was approved by the ethical committee of the Weizmann Institute. Participants were remunerated on an hourly basis. The participants were divided arbitrarily into the three experimental groups described below.

### Experimental protocol

**Figure 1:**
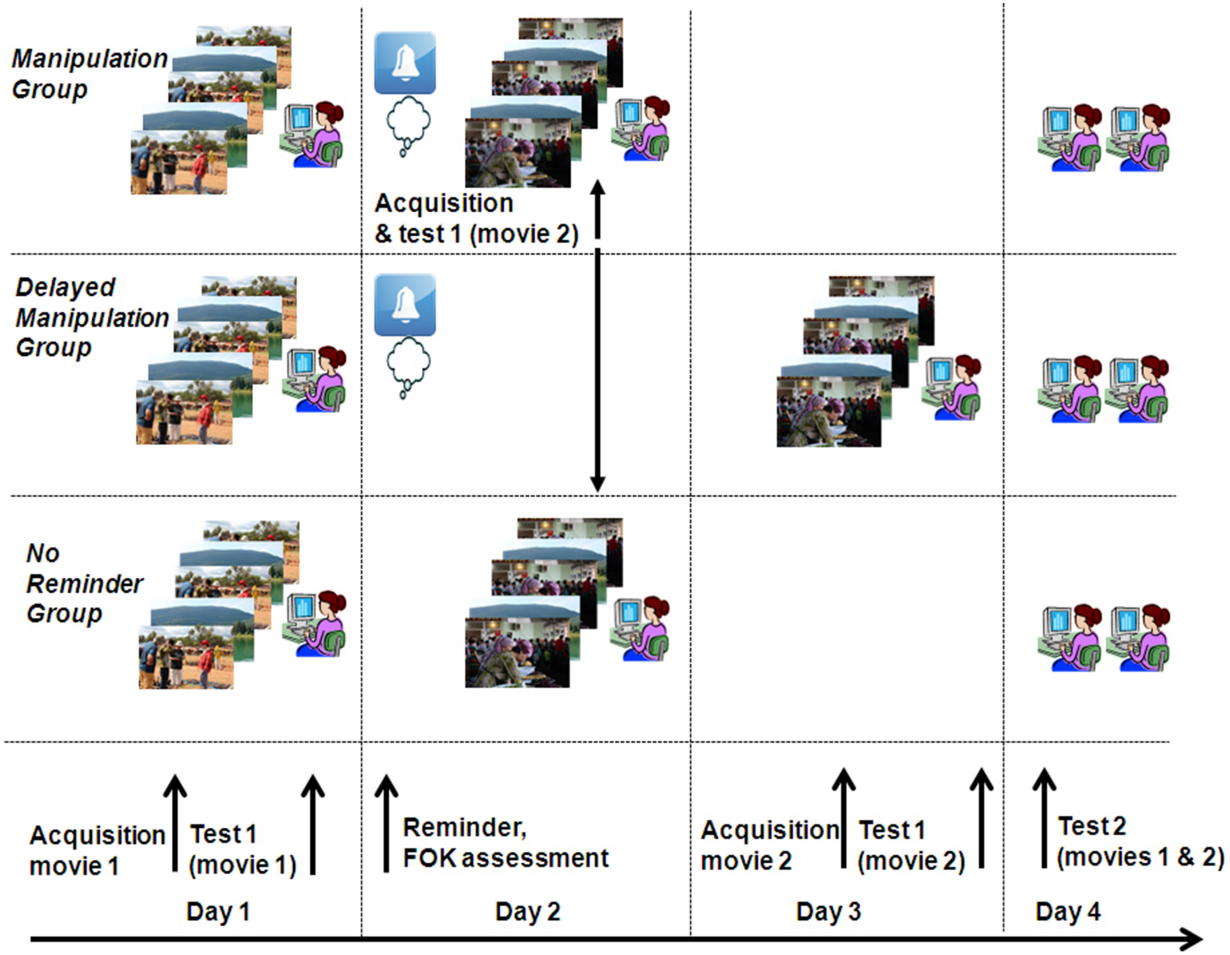
**Experimental design**. Participants from all three groups watched a movie (movie 1) and were subsequently tested on their memory of it (test 1). On day 2, participants of the *Manipulation* and *Delayed Manipulation Groups* were provided with reminders regarding scenes from movie 1, to which they were asked to rate their feeling of knowing (FOK). Immediately after the reminder phase (*Manipulation Group*) or on day 3 (*Delayed Manipulation Groups*) or devoid of the reminder (*No Reminder Group*) participants were presented with a second movie (movie 2) and tested on it. Finally, on day 4, all participants were tested again for their memory of movies 1 and 2 (test 2).

The experimental protocol spanned 4 days, each starting with written instructions explaining the tasks at hand (see Figure 1 for experimental scheme). On Day 1, all participants watched one of two Hebrew speaking documentary films on a computer screen supplemented with earphones (movie A, 5 min, *n* = 38; movie B, 7 min, *n* = 39). Both films depicted non-dramatic, emotionally neutral content, and included several scenes within the context of a central theme. One movie portrayed a news interview with a martial arts expert, who explained and demonstrated self-defense techniques. The other movie involved a job interview taken by two young men, displaying different interviewer prototypes. The movie (*first episode*) was presented three times intercalated with 1-min displays of tranquil nature scenes. Five minutes after the last presentation, memory performance for the learned movie was tested using a computerized questionnaire, in which 40 questions taxed occurrences of scenes from the movie (test 1). The questions probed memory for details about specific scenes or characters, such as: “What does Jeanne take with her to the interview?” or “What are Joe's expectations of the proposed job?” Four possible answers were presented for each question, from which only one was correct. Responses were provided by clicking A–D on the keyboard. After replying to each question, participants rated their confidence level regarding the perceived correctness of their response by pointing with the computer mouse on a visual-analog scale (VAS) presented on screen spanning continuously from low to high with 5 intermediate ticks. No feedback was provided to participants about their response.

On day 2, a reminder phase was carried out, aiming to reactivate memory for the film presented on day 1. The manner in which memory reminders are presented may significantly influence the long-term fate of the probed memories (Hupbach et al., 2007; Forcato et al., 2009a). It is well established that reactivating memories by means of memory tests can in fact strengthen those memories (Carpenter et al., 2008; Karpicke and Roediger, 2008), and block the influence of immediate interference (Potts and Shanks, 2012). Since our aim was reactivating the original memories without strengthening them, we built on previous reconsolidation studies that during the reminder phase exposed participants to partial retrieval cues (Forcato et al., 2007, 2009a,b; Hupbach et al., 2007), which possibly promote memory labilization by preventing the reinforcement of retrieved information (Pedreira et al., 2004; Forcato et al., 2009a). We thus chose to reactivate the learned films by providing textual instructions that served as general retrieval cues (Mendelsohn et al., 2008, 2009, 2010), followed by instructions to rate FOK levels of the retrieval attempt on a continuous VAS. The textual reminder cues included 20 sentences that were presented on screen, each referring to a selected occurrence from the movie presented on day 1. Unlike the memory test questions administered on day 1, which taxed specific details from particular scenes, each of the 20 reminder cues consisted of an instruction to recollect large sections from the movie, for example—“Please recollect the dialogue conducted during the interview scenes,” “Please recall the explanations about the various martial-art techniques shown in the film.” Each such sentence was presented for 20 s, during which participants were instructed to retrieve as much information as possible regarding the targeted scenes. For each reminder cue, they were prompted to self-appraise their FOK of the targeted occurrences on a continuous VAS with 5 intermediate ticks spanning from low to high FOK values, which were later translated to a scale of 0 (lowest rating) to 100 (highest rating).

Either 5 min after the reminder session (*Manipulation Group, n = 34, 16 females*) or without a preceding reminder (*No Reminder Group, n = 20, 9 females*) or a day later (*Delayed Manipulation Group*, *n* = 23, 10 females), participants watched a second short movie (*second episode*, movie B for those who watched movie A on day 1 and viceversa), in the same context as that of the first movie (i.e., same room, computer, experimenter, and experimental protocol). As in the encoding session on day 1, the movie was presented three times, followed by a 40-question test taxing memory for the movie's occurrences.

Finally, on day 4, participants of both groups were re-tested for their memory of both movies and provided corresponding confidence ratings (test 2). Memory for the first episode was tested before the second one. Once again, 40 questions were presented for each movie, for which participants were to choose the correct answer out of four possible options. Each question in test 2 was matched to a corresponding question in test 1, both targeting the exact same detail in the movie at each of the two tests but phrased somewhat differently. For example, test 1: “What is the first question the interviewer asked Eran” (one of the interviewed characters), test 2: “what is the first thing the interviewer said to Eran?”. This was done in order to avoid the possibility that instead of retrieving anew in test 2, participants would respond based on their memory for the questions and/or corresponding answers of test 1. Questions used for each of the two tests were assigned to each test in a counter balanced manner.

### Data analysis

Memory tests included 40 questions, each with four possible answers, only one of which was correct. Memory performance for each participant was computed as the proportion of correct responses in each questionnaire. Our main objective was to examine how post-reminder manipulations correspond to alterations in memory performance before and after the manipulation. To control for variability in baseline memory performance for test 1, administered immediately after encoding the first movie (see Supplementary Material), we computed a *memory strength* score for each participant, defined as the ratio between memory performance on day 4 and day 1 (i.e., memory performance of test2/test1), denoted as M_str_ = M4/M1, where M1 and M4 refer to memory performance on days 1 and 4, respectively. Note that since questions could be answered incorrectly in test 1 and correctly in the corresponding matched (but different) questions in test 2, *memory strength* scores higher than 1 are plausible. To corroborate the above analysis, we computed *memory strength* scores in an additional way, by computing the *difference* in memory performance on day 4 and day 1 instead of their ratio (*differential memory strength*). This analysis yielded very similar results, as reported in the Supplementary Material Section (see Results and Figure S1). For each group, average M_str_ scores were calculated and a subsequent One-Way ANOVA was carried out to test for potential differences among groups.

FOK scores were computed for each participant as the average ratings of the 20 reminder responses. In order to examine the relationship between FOK and *memory strength* scores or between FOK and learning memory performance measured on day 1 in test 1 within each group, we computed Pearson correlations between these measures for each group separately. To examine the differences between the groups' FOK vs. *memory strength* and FOK vs. pre-manipulation memory performance correlation slopes, analysis of covariance (ANCOVA) tests were performed to obtain a measure of the slope (β) for each group, which were subsequently compared among groups to test for differences in the slopes and intercepts of the regression lines (i.e., group × slope interactions). Since FOK scores were given in response to general retrieval cues targeting whole scenes and the memory tests taxed specific scene details, specific FOK ratings could not be attributed to particular memory items and directly compared.

## Results

**Figure 2:**
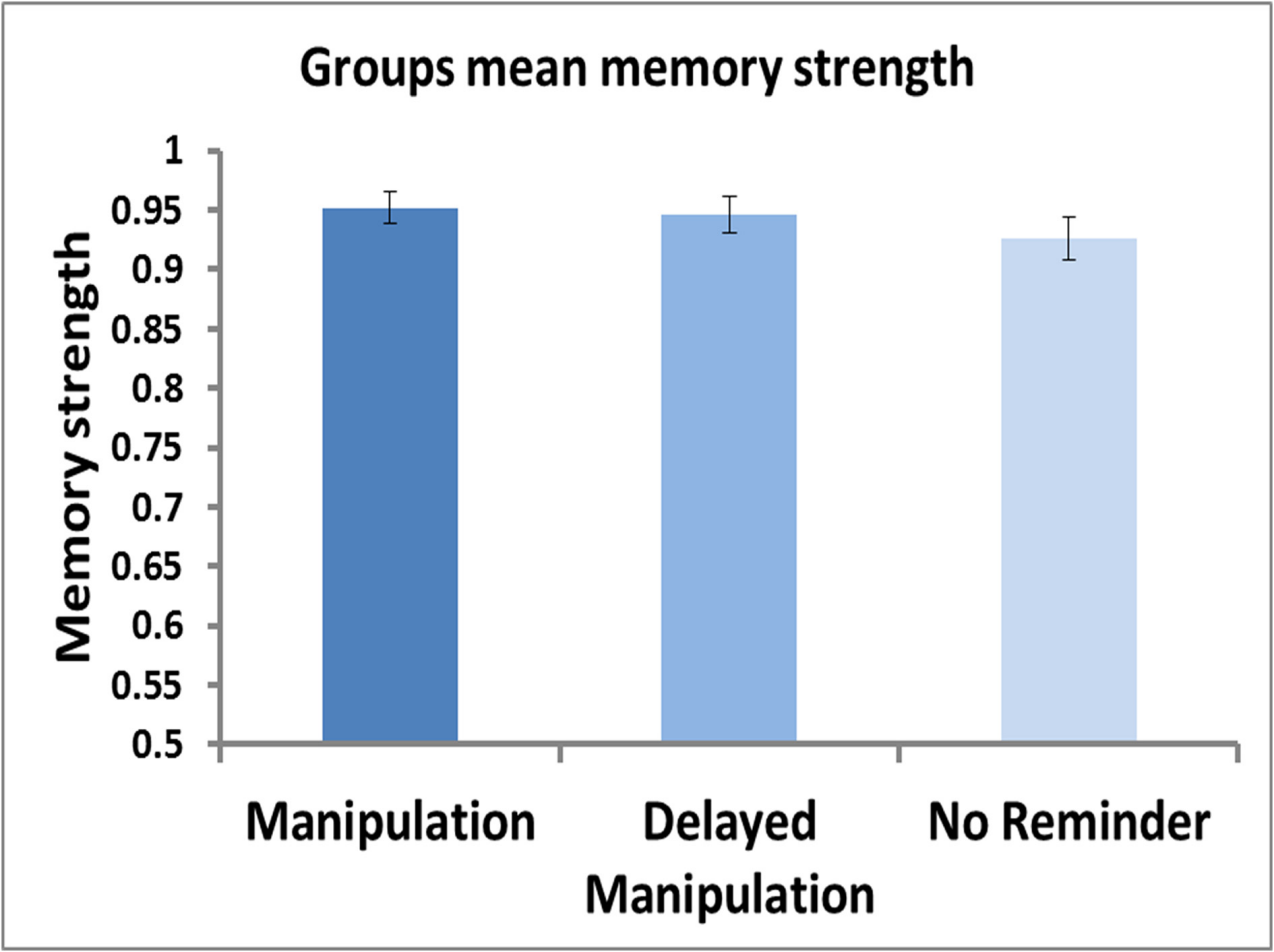
**Group means of memory strength**. Mean memory strength, calculated as the ratio of test2/test1, shown for each of the three groups (Manipulation Group, Delayed Manipulation Group, and No Reminder Group). No significant differences were found between the 3 groups [One-Way ANOVA: *F*_(2, 74)_ = 0.75, *p* = 0.48].

Testing for postulated reconsolidation effects among the three groups regardless of metamemory judgments did not yield significant findings, as mean memory strength scores for movie 1 did not differ among the three groups (Figure 2; One-Way ANOVA: *F*_(2, 74)_ = 0.75, *p* = 0.48). Thus, the absence of a reminder or the timing of post-reminder new learning material, were not sufficient to affect *memory strength* differences as assessed by average group performance irrespective of FOK ratings.

**Figure 3:**
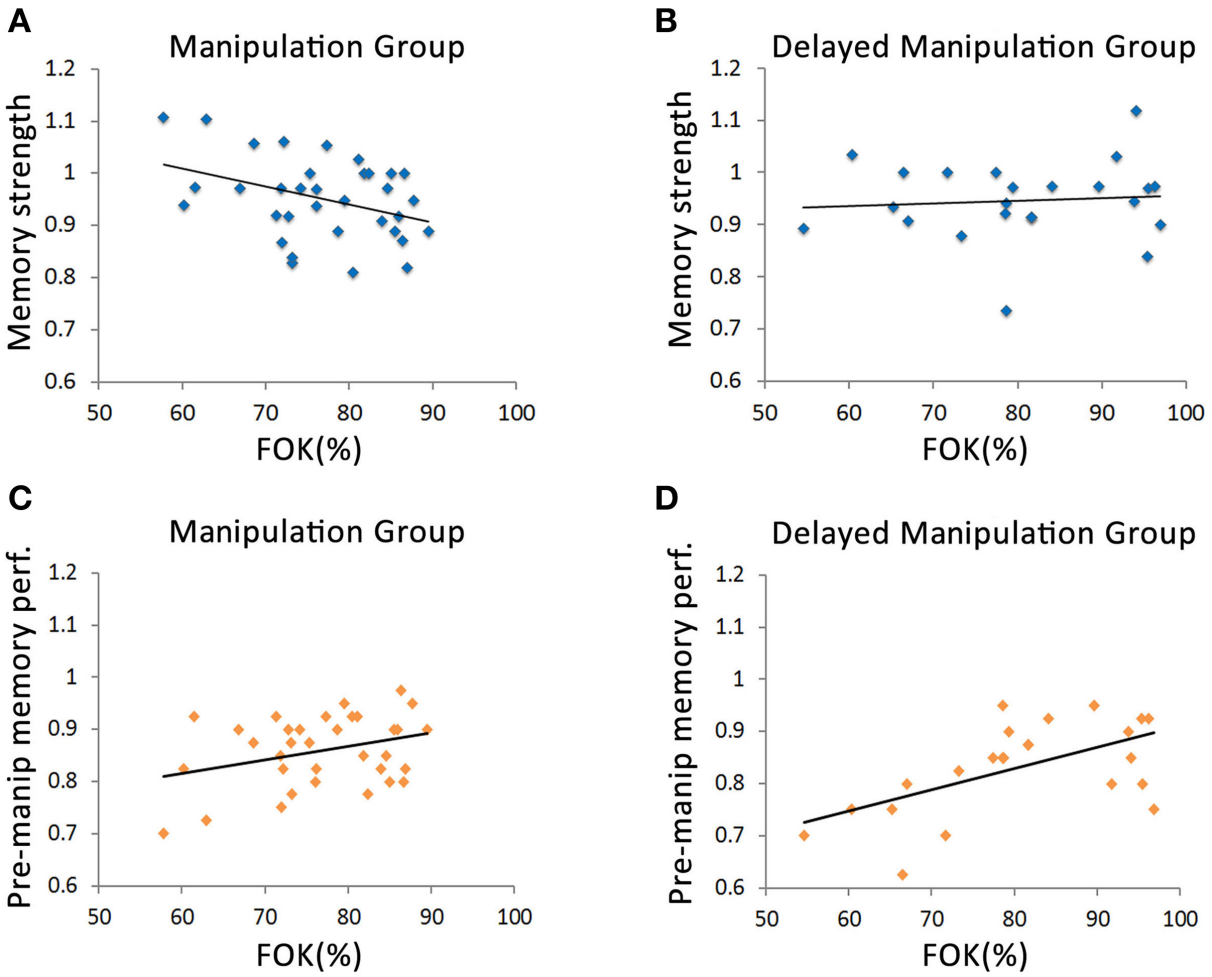
**Inter-participant correlations between FOK and Memory performance. (A)** Scatterplot of memory strength vs. FOK for Manipulation Group, where movie 2 was learned immediately after reactivation of movie 1. The plot indicates a negative correlation between FOK and memory strength (*r* = −0.38, *p* < 0.05). **(B)** Scatterplot of memory strength vs. FOK for the Delayed Manipulation Group, for which movie 2 was learned 1 day after reactivation of movie 1, demonstrating that memory strength is not correlated with FOK levels (*r* = 0.09, *p* = 0.69). The correlation slopes significantly differed between Manipulation Group and Delayed Manipulation Group [ANCOVA *F*_(3, 53)_ = 4.02, *p* = 0.05]. **(C–D)** Scatterplots of pre-manipulation memory performance (test 1) vs. FOK for the Manipulation Group **(C)** and Delayed Manipulation Group **(D)**. Both groups exhibited positive correlations between these two measures (Manipulation Group: *r* = 0.33, *p* = 0.055; Delayed Manipulation Group: *r* = 0.575, *p* < 0.05). The correlation slopes of Manipulation Group and Delayed Manipulation Group did not differ from one another [ANCOVA *F*_(3, 53)_ = 0.62, *p* = 0.43].

The primary aim of this study was to examine the relationship between metamemory evaluations of reminded information and the memory's susceptibility to post-reminder manipulations. We thus inspected the relationship between the average FOK rating of each of the participants measured upon reactivation of movie 1 and their corresponding *memory strength* scores. Specifically, Pearson correlations were computed between FOK and *memory strength* scores across participants for each group separately (Figures 3A,B). The *Manipulation Group*, which watched movie 2 immediately after the reminder-FOK assessment stage of movie 1, showed a negative correlation between *memory strength* and FOK, so that the lower their FOK was, the higher was their final *memory strength* score (*r*_manip_ = −0.38, *p* < 0.05, Figure 3A). In other words, the lower the FOK ratings for reactivated memory items, the less those memories were influenced by new learning material. This was true, however, only when new information was learned immediately after the reminder phase, as the *Delayed Manipulation Group*, that underwent *manipulation* a day after the reminder phase, displayed no meaningful correlation between FOK and *memory strength* (*r*_delay_ = 0.09, *p* = 0.69, Figure 3B). An analysis of covariance test (ANCOVA) yielded a significant difference between the slopes of these two groups (*F*_(3, 53)_ = 4.02, *p* = 0.05) demonstrating a clear divergence in memory performance vs. FOK correlations. Corroborating these results, correlating the FOK scores with the *difference memory strength* (i.e., calculating memory change by subtracting instead of dividing test 2 from test 1 scores) yielded similar results (*Manipulation Group: r*_manip_ = 0.38, *p* < 0.05, *Delayed Manipulation Group*: *r*_delay_ = −0.05, *p* = 0.43; ANCOVA—*F*_(3, 53)_ = 3.53, *p* = 0.06; see Figures S1A,B).

To rule out the possibility that these correlations were not related to the manipulation phase, we also measured the correspondence between FOK judgments and original memory performance (i.e., prior to the manipulation phase), by computing the correlation between memory performance scores of participants obtained at test 1 and corresponding FOK assessments taken on day 2. As might be expected (Sacher et al., 2009), memory performance and FOK assessments (assessed prior to the presentation of movie 2) were positively correlated in both the *Manipulation Group* (*r* = 0.33, *p* = 0.055, Figure 3C) and *Delayed Manipulation Group* (*r* = 0.575, *p* < 0.05; Figure 3D). Thus, prior to the manipulation phase (i.e., before immediate or delayed post-retrieval presentation of movie 2), no apparent differences were detected between groups in the relationship between memory performance and FOK (ANCOVA—*F*_(3, 53)_ = 0.62, *p* = 0.43). Similarly, average FOK ratings were not significantly different between the two groups (*Manipulation Group* mean, SE: 76.8, 1.46%, *Delayed Manipulation Group*: 80.5, 2.5%, *t*-test: *p* = 0.21). These findings strengthen the notion that post-retrieval changes in memory performance are influenced by metamemory assessments upon reactivation and the timing of post-reminder manipulation.

Taken together, these results demonstrate the involvement of metamemory judgments in the long-term fate of reactivated memories, so that the lower one's FOK judgments during reactivation, the lower the chances that immediate learning of new information will degrade those reactivated memories.

## Discussion

Our results demonstrate a role of metamemory processes in the fate of long-term memories such that one's perceived memory strength during retrieval attempts of naturalistic episodes can predict whether and to what extent those memories might change. Specifically, we show that the perceived memory availability of a previously learned film (assessed by FOK ratings), if immediately followed by acquisition of new material, is proportional to long-term deterioration in memory performance. To construe the relation between metamemory judgments—probed here by FOK assessments—and the fate of long-term memory, it is useful to appreciate what FOK assessments signify. Two leading models that aim to explain FOK judgments are the *cue-familiarity* model (Reder and Ritter, 1992) and the *accessibility account* (Koriat, 1993). The cue-familiarity model accounts for rapid preliminary FOK, where the familiarity of the cue (or pointer), but not the actual memory (target), serves as the basis for making FOK judgments (Metcalfe et al., 1993). Accordingly, FOK judgments are made by assessing the familiarity of presented information, which is often, though not always, correlated with accurate memory retrieval (Schnyer et al., 2004; Sacher et al., 2009; Hertzog et al., 2010). The accessibility account of FOK states that metamemory judgments are based on any partial available information relevant to targeted items, not necessarily limited to those elicited by the cue (Schacter and Worling, 1985; Koriat, 1993; Pannu and Kaszniak, 2005).

A model that combines the cue-familiarity and accessibility accounts (Koriat and Levy-Sadot, 2001), suggests that cues are initially assessed for familiarity, whereby low familiarity can terminate attempts to retrieve memory items, and high familiarity will initiate further search of the targeted memory. This model contends that monitoring and retrieval are two components of a single process, during which FOK judgments can be formed based on the overall availability of targeted items (Koriat, 1993, 1994). FOK judgments are thus generated by the accumulation of correct and incorrect partial information accessible during the monitoring and retrieval process (Koriat, 1993, 1994), and are typically positively correlated with memory accuracy (Koriat, 1994; Sacher et al., 2009), a notion that coincides with our findings. Based on the above, our working assumption is that FOK rates reflect the extent of accessible information pertinent to the target memory.

As mentioned above, it was not merely the time-frame of reminder-immediate new learning that engendered a change in long-term *memory strength*, but also the extent of perceived memory during the reminder phase. The lower the memory reactivation of the original film—expressed by FOK ratings—the less the immediate new learning affected its long-term retention. The notion that among the many memory traces that may be retrieved at a given point, the highly reactivated memories are those that are most susceptible to modifications resonates with previous animal and human studies. In rodents and fish, the dominant association that had more control over behavior out of those paired with a retrieval cue was the one that reconsolidated (Eisenberg et al., 2003). A similar effect was recently observed for hippocampal-dependent memory in humans, whereby newly presented visual contexts were “bound” with previously learned items, yet only when those items represented the dominant memory traces, indicating that memory modification is related to the extent of reactivation (Bridge and Voss, 2014). In a recent episodic memory study in humans, items encoded during a museum tour that were subjectively reported to be well remembered were more prone to incorporation of false information presented during the reminder phase than less recollected memories (St. Jacques and Schacter, 2013). This implies that although highly reactivated memories are generally recalled accurately and vividly, they are also more sensitive to post-retrieval manipulations. In line with the aforementioned studies, we propose that the degree of FOK ratings in response to retrieval cues, representing memory reactivation levels, is one of the predictors of the memory's susceptibility to post-reactivation manipulation.

When considering the change in average memory performance for the content of the original movie, disregarding inter-subject variations in metamemory ratings, our results showed no difference among all three groups. Thus, on average, all groups showed a similar reduction in memory performance between initial and final tests, whether they received a reminder that was immediately followed by a second learning phase (*Manipulation* Group), underwent the new learning stage 1 day after the reminder (*Delayed Manipulation* group), or learned the new episode devoid of a reminder (*No Reminder* group). That the reminder, postulated to reactivate the cued memory, did not stimulate memory facilitation (Gisquet-Verrier and Riccio, 2012) may be explained by the fact that we provided a single reminder only, unlike other studies that used either multiple reminders or stronger ones as detailed below. For instance, it was demonstrated that memory enhancement for paired syllables required at least two consecutive cue reminders following the presentation of an initial reminder (Forcato et al., 2011). In another study, three consecutive reminders in the form of instructed retrieval of the learning material, similar to the reminder phase in our paradigm, boosted memory strength whereas a single retrieval reminder phase was ineffective in enhancing memory performance (Wichert et al., 2013). Memory facilitation was also shown to occur when using more potent reminders than in our study in the form of a memory test (Potts and Shanks, 2012). In addition, whereas the reminder phase included general retrieval cues associated with the target memory, reminders that facilitate memory might require substantial resemblance to the encoded material (St. Jacques and Schacter, 2013).

An additional factor that may explain the lack of an overall average group reconsolidation effect in our study might be the type of post-reminder manipulation used. Specifically, the post-reminder learning material applied here did not pose direct interference to the reminded material, as in some declarative memory studies demonstrating reconsolidation. For example, providing reminders to A-B pairs of previously learned non-sense syllables, followed immediately by the encoding of a new set of A-C syllable pairs, yielded higher error rates in the memory for the original list than those of control groups with either delayed or no interference (Forcato et al., 2007). A similar effect was found for memory of lists of objects, whereby providing a reminder for the original list immediately before learning a new object list, increased intrusions of new items (Hupbach et al., 2007). Tampering with long-term episodic memory of a movie was achieved by presenting false information related to the originally encoded material immediately after reactivating it (Chan and LaPaglia, 2013). Introducing interfering information during the postulated malleable state does not however seem to be a pre-requisite for reconsolidation, as encoding new post-reminder information unrelated to the original material can also lead to its reconsolidation (Schwabe and Wolf, 2009). A study that tested boundary reconsolidation conditions of declarative memories in humans reported weak or null reconsolidation effects in a reactivation-interference protocol using sets of pictures as memoranda (Wichert et al., 2011). Importantly, similar to our paradigm, the post-reminder learning material in that study did not pose a direct interference to the original information, a fact that might explain the weak or lack of group reconsolidation effects. All considered, it is possible that had we presented post-reminder learning material that directly interfered with the memory of the original film, a more robust reconsolidation effect may have been set in motion regardless of the extent of memory reactivation.

To summarize, we show that the subjective degree of episodic memory reactivation may determine, under conditions that are postulated to favor reconsolidation, the long-term fate of those memory representations. This finding extends the current view regarding the conditions assumed to promote reconsolidation of long-term naturalistic memories, suggesting a significant role for the retriever's perception of the memory's accessibility on its final outcome. These findings could be taken into consideration in reconsolidation protocols in which altering memory, and particularly of episodic nature, is desirable.

### Conflict of interest statement

The authors declare that the research was conducted in the absence of any commercial or financial relationships that could be construed as a potential conflict of interest.
