# Supplementary material for: Metamemory ratings predict long-term changes in reactivated episodic memories
Source: Front Behav Neurosci. 2015 Feb 9;9:20. doi: 10.3389/fnbeh.2015.00020 (PMC4321599; doi:10.3389/fnbeh.2015.00020)
Supplement: Supplementary file 1 [file DataSheet1.PDF]

Supplementary Material

Memory performance at day 1

Participants' memory performance was measured during test 1 after 3 learning cycles.
Means, variability and standard deviations of memory performance on test 1 for each of the 3 groups was:
Manipulation Group: mean=0.86, range = 0.7–0.98, standard deviation = 0.067.
Delayed Manipulation Group: mean=0.83, range = 0.62-0.95, standard deviation=0.088
No Reminder Group: mean=0.83, range = 0.7-0.92, standard deviation=0.063.

Memory strength

Memory strength was computed by calculating the ratio of memory performance of episode 1 measured during test 2 on day 4 to the memory performance of the same episode measured at test 1 on day 1: M_{str}=M4/M1. In order to examine whether the obtained results were not specific to the method of calculation, we employed a *differential memory strength* score by computing the difference between M1 and M4: M_{str}=M1 minus M4. Note that the 'difference' method reverses the direction of *memory strength* so that high values now indicate larger decreases in memory performance than low values.

Based on the 'difference' method, Pearson correlations were computed between FOK and *differential memory strength* scores across participants for each group separately (Figure S1A-B). Similar to the reported 'ratio' method, the *Manipulation Group*, which was presented with movie 2 immediately after the reminder of movie 1, demonstrated a positive correlation between *differential memory strength* and FOK, so that the higher their FOK was, the higher was their memory difference ($r_{manip}=0.38$, $p<0.05$, Figure S1A). In contrast, the *Delayed Manipulation Group*, presented with movie 2 one day after the reminder phase, displayed no meaningful correlations between FOK and *differential memory strength* ($r_{delay}=-0.05$, $p=0.43$, Figure S1B). An analysis of covariance test (ANCOVA) yielded a trend of difference between the slopes of these two groups ($F_{3,53}=3.53$, $p=0.06$) demonstrating a trend of divergence in memory difference vs. FOK correlations.

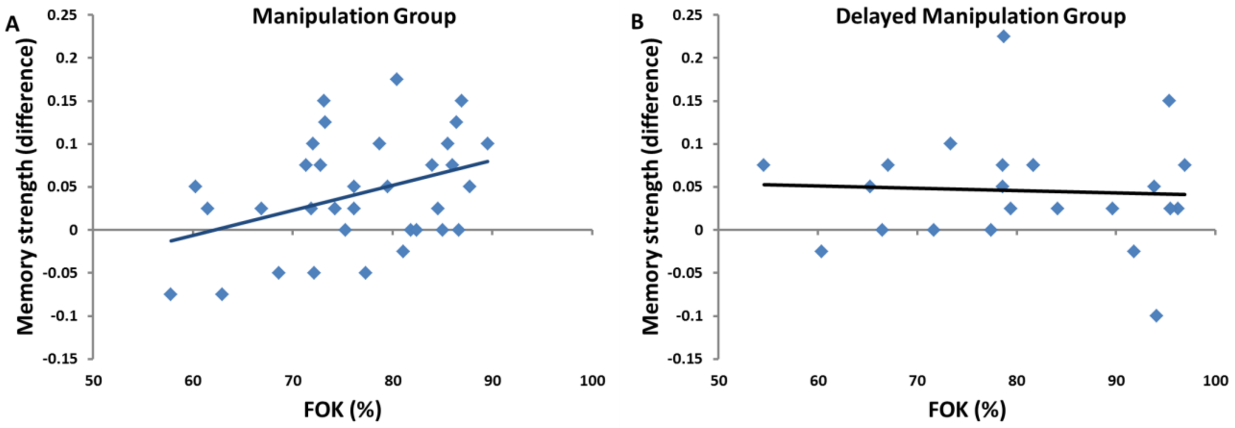

Figure S1. Scatterplots of *differential memory strength* calculated using the 'difference' method (see above) vs. FOK for *Manipulation Group* (A) and *Delayed Manipulation Group* (B). Corroborating the results reported using the original 'ratio' calculation (Figure 3), the *Manipulation group* showed a positive correlation between FOK and decreases in *memory strength* (r=0.38, p<0.05), whereas the *Delayed Manipulation Group* showed no such effect (r=-0.05, p=0.43). The correlation slopes exhibited a trend of difference between *Manipulation Group* and *Delayed Manipulation Group* (ANCOVA F(3,53)=3.53, p=0.06).
